# Remodeling of extra-bronchial lung vasculature following allergic airway inflammation

**DOI:** 10.1186/1465-9921-9-18

**Published:** 2008-02-08

**Authors:** Kristina Rydell-Törmänen, Lena Uller, Jonas S Erjefält

**Affiliations:** 1 Div. Vascular and Airway Research, Dept. Experimental Medical Science, Lund University, Lund, Sweden

## Abstract

**Background:**

We previously observed that allergen-exposed mice exhibit remodeling of large bronchial-associated blood vessels. The aim of the study was to examine whether vascular remodeling occurs also in vessels where a spill-over effect of bronchial remodeling molecules is less likely.

**Methods:**

We used an established mouse model of allergic airway inflammation, where an allergic airway inflammation is triggered by inhalations of OVA. Remodeling of bronchial un-associated vessels was determined histologically by staining for α-smooth muscle actin, procollagen I, Ki67 and von Willebrand-factor. Myofibroblasts were defined as and visualized by double staining for α-smooth muscle actin and procollagen I. For quantification the blood vessels were divided, based on length of basement membrane, into groups; small (≤250 μm) and mid-sized (250–500 μm).

**Results:**

We discovered marked remodeling in solitary small and mid-sized blood vessels. Smooth muscle mass increased significantly as did the number of proliferating smooth muscle and endothelial cells. The changes were similar to those previously seen in large bronchial-associated vessels. Additionally, normally poorly muscularized blood vessels changed phenotype to a more muscularized type and the number of myofibroblasts around the small and mid-sized vessels increased following allergen challenge.

**Conclusion:**

We demonstrate that allergic airway inflammation in mice is accompanied by remodeling of small and mid-sized pulmonary blood vessels some distance away (at least 150 μm) from the allergen-exposed bronchi. The present findings suggest the possibility that allergic airway inflammation may cause such vascular remodeling as previously associated with lung inflammatory conditions involving a risk for development of pulmonary hypertension.

## Background

Allergic airway inflammation is known to be associated with persistent inflammation and tissue remodeling, such as subepithelial fibrosis, smooth muscle thickening and increased vascularity in the bronchial circulation [1-3]. Most of our knowledge of remodeling in asthma emanates from studies of bronchial biopsies involving the large airways. However, asthma is not only a large airway disease, but also affects other parts of the lung, notably the small airways and possibly the bronchial-associated blood vessels [4,5]. Partly in agreement with findings in severe asthma [5] we have previously shown [6] that large blood vessels adjacent to mouse allergen-exposed bronchi are inflamed and exhibit vascular remodeling, a feature recently also described in asthma [7].

In the current literature there are only a few descriptions of pulmonary vascular remodeling following allergic airway inflammation. By contrast, remodeling of these vessels is an accepted feature of several other diseases such as systemic scleroderma [8], idiopathic pulmonary fibrosis [9] and COPD [10,11]. The mechanism behind vascular remodeling may vary with disease, but the histological appearances appear to be similar in nature [12]. In a previous paper [6], we reported that remodeling of the large pulmonary vessels was as prominent as the bronchial remodeling in a mouse model of airway allergic inflammation. For example, similar increases in smooth muscle mass, collagen synthesis and proliferation occurred in both airways and adjacent large vessels, a phenomenon tentatively explained by a spill-over of inflammatory mediators released in the adjacent bronchial tissues.

Here we report that vascular remodeling changes are also present in smaller extra-bronchial (pulmonary) solitary vessels, and include the occurrence of phenotypic changes in partially muscularized blood vessels and appearance of perivascular myofibroblasts.

## Methods

### Model of allergic inflammation

Female BALB/C mice (15–20 grams, MoB A/S, Ry, Denmark) were sensitized day 0 with ovalbumin (n = 10, 10 μg OVA (grade III, Sigma, St Louis, MO) + 1 mg AlOH_3 _i.p.) or saline (n = 7), followed by daily 30 min 1% OVA aerosol exposures days 14–20, according to a validated protocol [13]. Day 21 the animals were sacrificed and lung tissue specimens were processed for cryostat (eosinophil-staining) and paraffin sectioning (IHC- and TUNEL-staining) [13]. For all immunohistochemical staining a standard protocol was used, as previously described [6]. Briefly antibody was applied onto the section in appropriate dilution, incubated over night in 4°C, washed the next day and incubated with secondary antibody (45 minutes in room temperature). All animal protocols were approved by the local ethics committee (Malmö/Lund, Sweden).

### Tissue eosinophilia and cell turnover

The presence of an allergic airway inflammation was determined as lung tissue eosinophilia, (enzyme-histochemical staining for eosinophil peroxidase, EPO [14]). Proliferation was detected by immunohistochemical staining for the proliferation antigen PCNA (U7032, Dako A/S, Glostrup, Denmark). Apoptosis was detected by TUNEL technique, as previously described [15], and visualized by an anti-DIG antibody combined with New Fuchsine (D5105 and K698, both Dako). Eosinophilia and cell turnover was quantified in digital images as labelled cells/area (mm^2^).

### Remodeling

An antibody against α-smooth muscle actin (α-SMA, 1:300, clone 1A4, Sigma) was used to visualize smooth muscle cells (SMC). Collagen synthesis was assessed by staining for procollagen I (as previously described [6], PINP, 1:200, a kind gift from Professor Juha Risteli, Oulo University, Oulo, Finland). Myofibroblasts were defined as solitary cells co-positive for procollagen I and α-SMA. An anti-von Willebrand factor antibody was used to visualize endothelial cells (1:640, A0082, Dako). Proliferating SMC and endothelial cells were visualized by double-staining with the proliferation marker Ki67 (M7249, 1:200, clone TEC-3, Dako).

### Quantification and statistics

Histologic analysis was preformed as previously described [6]. Briefly, high-resolution digital images (3 images per section) were obtained in a random fashion. Transversally cut blood vessels within the sections (excluding any bronchial-associated vessels) were analyzed after being divided into subgroups depending on size into small (≤250 μm in perimeter) and mid-sized (250–500 μm in perimeter) vessels. All vessels were non-bronchial associated (solitary), defined as being ≤150 μm distant from any bronchi. Quantification was done as previously described [6], briefly the length of the vascular basement membrane (BM) was assessed by manual cursor tracing and the labelled area/number of cells was quantified and correlated to the length of the BM. All quantifications were made in a blinded fashion, and the Wilcoxon Signed-ranks test was used for statistical analysis (Analyze It™, Analyze-it Software Ltd, Leeds, UK). Data are given as mean values ± SEM, and p < 0.05 was considered statistically significant.

## Results

### Eosinophilia and cell turnover

Seven days of allergen exposure initiated a prominent eosinophilia, with both perivascular and peribronchial distribution. The total number of eosinophils in sections were significantly increased in allergen exposed animals (231 ± 42 cells/mm^2^) compared to controls (63 ± 31 cells/mm^2^, p < 0.05). Notably, perivascular eosinophilia was present around both small and mid-sized solitary blood vessels. The overall cell turnover in lungs increased following allergen exposure, both the number of proliferating and apoptotic cells increased significantly. The number of proliferating cells increased from 2.9 ± 0.6 cells/mm^2 ^in controls to 18.7 ± 2.8 cells/mm^2 ^following allergen challenge (p < 0.05), whereas the number of apoptotic cells increased from 12 ± 2.4 cells/mm^2 ^in controls to 26 ± 5.7 (p < 0.05).

### Endothelial cell turnover following allergen exposure

**Figure 1:**
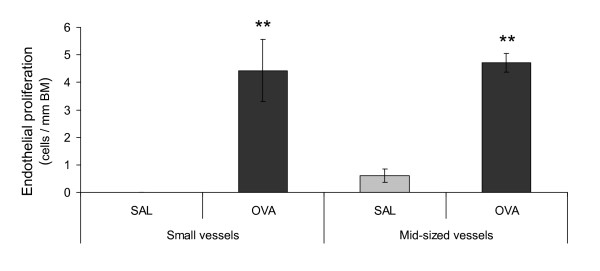
**Endothelial proliferation increased following allergic airway inflammation**. The number of proliferating endothelial cells in small and mid-sized blood vessels increased following allergen challenge. Proliferation was detected using the proliferation-marker Ki67. A base line proliferation was present also in controls, however the number was very low and when correlated to the length of the basement membrane (BM), the values closed in on zero. The data are given as mean ± SEM and compared against control using the Wilcoxon Signed-ranks test, * indicates p < 0.05.

The number of proliferating endothelial cells in mid-sized blood vessels increased significantly in response to allergen (Figure 1), and the number of proliferating endothelial cells in small vessels displayed a similar increase (Figure 1). The apparent lack of proliferating cells in controls is somewhat misleading, as proliferating cells was seen in controls. However the baseline proliferation was very low and correlation to the length of the BM results in a very low value. No increase of the number of apoptotic endothelial cells was detected in either mid-sized or small blood vessels following allergen exposure (data not shown).

### Vascular smooth muscle remodeling

**Figure 2:**
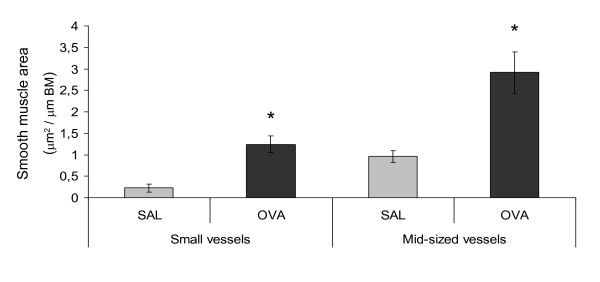
**Smooth muscle mass increase in both small and mid-sized blood vessels following allergen challenge**. The area of vascular smooth muscle increased after allergen exposure, in small vessels approximately 5 times and in mid-sized approximately 3 times. Smooth muscle was detected by labelling with α-smooth muscle actin, and the positively stained area measured by digital image analysis and correlated to the length of the basement membrane (BM). The data are given as mean ± SEM and compared against control using the Wilcoxon Signed-ranks test, * indicates p < 0.05.

**Figure 3:**
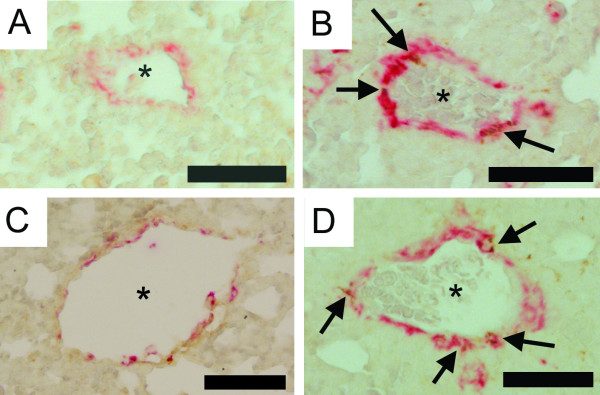
**Photomicrographs illustrating vascular remodeling following 7 days of allergen exposure**. Vascular remodeling involves smooth muscle (α-smooth muscle actin: red), fibroblasts (procollagen I: brown) and myofibroblasts (here defined as solitary cells co-positive for α-smooth muscle actin and procollagen I: co-positive), in both small solitary (A-B) and mid-sized solitary (C-D) vessels. In comparison with controls (A and C) vessels from OVA exposed animals (B and D) show a significantly increased smooth muscle area as well as increased number of myofibroblasts (arrows) and procollagen I-producing cells. Vascular lumen is indicated by stars. Scale bar represents 50 μm.

The perivascular smooth muscle area of mid-sized vessels was significantly increased in animals exposed to allergen (Figures 2 and 3). The increase was at least partially due to hyperplasia, since the number of proliferating smooth muscle cells also increased (1.7 ± 0.6 in controls to 10.8 ± 0.6 cells/mm in allergen exposed animals (p < 0.01)), whereas the number of apoptotic smooth muscle cells did not change (data not shown). Small blood vessels displayed again a similar increase as the mid-sized; the perivascular smooth muscle area increased significantly following allergen exposure (Figures 2 and 3), and thus causing muscularization of partially muscularized blood vessels. The number of proliferating smooth muscle cells was significantly increased in animals exposed to allergen, increasing from 0.1 ± 0.4 cells/mm in controls to 11 ± 0.9 in allergen exposed animals (p < 0.01), the number of apoptotic cells did not change (data not shown).

### Alteration of collagen synthesis

**Figure 4:**
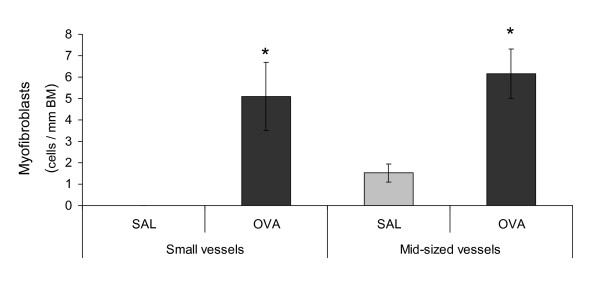
**Significant increase in myofibroblasts numbers following allergen challenge**. The number of myofibroblasts (defined as solitary cells co-positive for α-smooth muscle actin and procollagen I) increased significantly following allergen exposure. Occasional smooth muscle cells may produce procollagen I; however smooth muscle cells are not normally solitary. Sporadic myofibroblasts were also visible before allergen challenge, but when correlated to the length of the basement membrane (BM) the values are very small and closed in on zero. The data are given as mean ± SEM and compared against control using the Wilcoxon Signed-ranks test, * indicates p < 0.05.

In sections from allergen exposed animals, the number of procollagen I-positive cells increased significantly around middle-sized blood vessels (p < 0.05), from 1 ± 0.4 in controls to 4.1 ± 0.4 cells/mm in allergen exposed animals. A similar increase was detected in the number of myofibroblasts, defined by co-positive for α-SMA and procollagen I (Figures 3 and 4). In contrast to middle-sized blood vessels, no significant increase in the number of procollagen I-positive cells around small blood vessels was found. Nevertheless, the number of myofibroblasts was significantly increased around small blood vessels (Figures 3 and 4).

## Discussion

The present study demonstrates remodeling in small to mid-sized solitary vessels in the lungs of allergic mice challenged intra-bronchially with allergen. The remodeling characteristics observed in this study were similar to changes previously described in large bronchial-associated vessels [6], with a few exceptions. We thus made the additional observation here that small, previously poorly or partially muscularized blood vessels transformed to a more muscularized phenotype (increased perivascular smooth muscle mass), accompanied by the appearance of myofibroblasts. The increased number of proliferating SMC was similar in the large and mid-sized blood vessels but greatly enhanced in small vessels. In contrast, the number of procollagen I producing cells was not increased in small vessels, yet increased in mid-sized (this study) and large vessels (5). Taken together our results suggest that allergic airway inflammation in mice involves size-dependent remodeling features in lung blood vessels irrespective of how close these vessels are to the allergen-exposed bronchi. The mean inner diameter of small solitary vessels displayed a slight reduction in allergen-exposed animals, whereas the external diameter did not change (data not shown). This alteration cannot be completely ruled out as an artefact; however the a more likely explanation is that increased smooth muscle reacts more strongly to the sacrificial procedure and thus decrease the diameter more than controls. This would be in accordance with the findings of Witzenrath [16], who found vessels from OVA-sensitized and -challenged mice to be hyper reactive.

The vessels were divided into subgroups depending on size and localization, due to practical reasons; the small solitary vessels (microvessels) were very difficult to exactly differentiate into arterioles or venules. Larger vessels can however be classified; the criteria is primarily based on localization and the presence of elastic lamina(s) and we have not been able to find a definition applicable for mice. Nevertheless, due to these criteria the "mid-sized solitary vessels" is defined as (post-capillary) veins and since they are located within the lung parenchyma they are most likely pre-septal veins. The vessels described in the previous study [6] is by this definition pulmonary arteries. Our results thus suggest that vascular remodeling occurs in both pre- and post-capillary vessels, as well as in the microvessels.

Exactly how the vascular remodeling is induced is not known; it has been speculated that lung vascular remodeling is induced by "spill over" of inflammatory mediators from the bronchi [5]. However, the present results, demonstrating marked remodeling effects in more remote pulmonary vessels some distance away (at least 150 μm) from the bronchi, may not support this notion. Thus the link between allergic bronchial processes and pulmonary vascular inflammation and remodeling remains elusive. Since the OVA used in the study is not endotoxin free, it cannot be ruled out that the vascular remodeling is caused by the endotoxin (alone or in combination with OVA), although this seems unlikely as studies utilizing sham-sensitized OVA-challenged animals, find no airway inflammation. More interestingly, it has recently been reported that chymase-positive mast cells are involved in the remodeling of the bronchial circulation of asthmatics [17]. The importance of eosinophils in the development of bronchial remodeling has also been highlighted recently [18], and future studies will have to show whether similar mechanisms may operate in the pulmonary circulation. Another possible explanation behind the vascular remodeling includes increased activity of local fibroblasts. The rather prompt appearance of myofibroblasts following allergen challenge in this study is intriguing. Furthermore, we observed that some myofibroblasts were localized between the basement membrane and smooth muscle layer. Thus we cannot exclude the possibility that migrating and proliferating fibroblasts have contributed to the present vascular remodeling effects. Indeed, a difference between cells from OVA-challenged animals and controls have been established in cultured fibroblasts, where cells from OVA-challenged animals demonstrates pro-fibrotic activities[19]. However the molecular regulation of this latter transformation has not yet been determined.

Although remodeling of the pulmonary circulation is a likely cause of pulmonary hypertension [8,9,20], asthma is not normally associated with pulmonary hypertension. However, an extensive literature search revealed some interesting material in this regard; Salako *et. al*. [21] reported transient pulmonary hypertension during an asthma attacks and several papers actually connect asthma and pulmonary hypertension/cor pulmonale [22-26]. Several of these clinical studies are old representing time periods when asthma was not as well treated as today. Today asthmatic inflammation is usually better controlled by drugs, speculatively reducing also the risk for development of serious vascular remodeling. Furthermore, the pulmonary vasculature exhibits a great dynamic ability to compensate for structural changes, which may result in clinical symptoms not showing until later stages of the disease and then only as very modest increases. This is indicated by reports of scleroderma patients displaying vascular remodeling even without clinical symptoms [12]. Also, it is of note that even small increases in the pulmonary pressure in COPD patients is associated with increased mortality [27]. Furthermore, our results highlights the involvement of inflammation in the development of pulmonary vascular remodeling, in accordance with previous publications [28,29], indicating a role of inflammation in the vascular remodeling associated with PAH.

It was recently shown that the pulmonary vasculature displays a hyper-reactive phenotype in response to for example serotonin [16], following allergic airway inflammation in animals. The authors did not speculate in any clinical implications, but the results suggest a direct connection between allergic airway inflammation and physiologic vascular responses in experimental animals. Tigani and co-workers [30], recently reported that vascular remodeling also occurs in Brown Norway rats following OVA-challenge, and similar changes are also present in asthmatic patients [7]. Based on the current results, we conclude that lung vascular remodeling is present in several animal models of airway inflammation, in both pre- and post capillary vessels, and potentially causes significant physiological effects. Based on the findings by Salako *et. al *[21] in asthma, and the likelihood that vascular remodeling is physiologically important in several other clinical conditions [8-11,20], we suggest that our findings may have some clinical relevance. However, it is important to recognize that the OVA-model used in this study is far from being equivalent to human disease [31] although it can be argued that similarly expressed lung vascular remodeling occurs in mice and humans.

## Conclusion

In summary, we have shown that, in our mouse model of allergic airway inflammation, vascular remodeling affects small, mid-sized and large blood vessels. The bronchial allergen challenges result in classical vascular remodeling as well as phenotypic changes particularly in the small pulmonary blood vessels. Our results suggest that allergic airway inflammation is accompanied by remodeling of the entire pulmonary circulation potentially increasing the risk for development of pulmonary hypertension.

## Competing interests

The author(s) declare that they have no competing interests.

## Authors' contributions

KRT designed of the study, played a major role in the acquisition, analysis and interpretation of data and drafted the manuscript. LU participated in the *in vivo*-procedures, analysis of data and writing the manuscript. JSE participated in the design of the study, the *in vivo*-procedures and writing of the manuscript. All authors read and approved the final manuscript.
